# Aerobic digestion reduces the quantity of antibiotic resistance genes in residual municipal wastewater solids

**DOI:** 10.3389/fmicb.2013.00017

**Published:** 2013-02-12

**Authors:** Tucker R. Burch, Michael J. Sadowsky, Timothy M. LaPara

**Affiliations:** ^1^Department of Civil Engineering, University of MinnesotaMinneapolis, MN, USA; ^2^Biotechnology Institute, University of MinnesotaSt. Paul, MN, USA

**Keywords:** antibiotic resistance genes, municipal wastewater treatment, aerobic digestion, class 1 integrons, qPCR

## Abstract

Numerous initiatives have been undertaken to circumvent the problem of antibiotic resistance, including the development of new antibiotics, the use of narrow spectrum antibiotics, and the reduction of inappropriate antibiotic use. We propose an alternative but complimentary approach to reduce antibiotic resistant bacteria (ARB) by implementing more stringent technologies for treating municipal wastewater, which is known to contain large quantities of ARB and antibiotic resistance genes (ARGs). In this study, we investigated the ability of conventional aerobic digestion to reduce the quantity of ARGs in untreated wastewater solids. A bench-scale aerobic digester was fed untreated wastewater solids collected from a full-scale municipal wastewater treatment facility. The reactor was operated under semi-continuous flow conditions for more than 200 days at a residence time of approximately 40 days. During this time, the quantities of *tet*(A), *tet*(W), and *erm*(B) decreased by more than 90%. In contrast, *intI1* did not decrease, and *tet*(X) increased in quantity by 5-fold. Following operation in semi-continuous flow mode, the aerobic digester was converted to batch mode to determine the first-order decay coefficients, with half-lives ranging from as short as 2.8 days for *tet*(W) to as long as 6.3 days for *intI1*. These results demonstrated that aerobic digestion can be used to reduce the quantity of ARGs in untreated wastewater solids, but that rates can vary substantially depending on the reactor design (i.e., batch vs. continuous-flow) and the specific ARG.

## Introduction

The resistance of pathogenic bacteria to antibiotic chemotherapy is a growing problem with significant consequences for public health. In the United States, methicillin-resistant *Staphylococcus aureus* (MRSA) infections lead to more fatalities than the combination of HIV/AIDs, Parkinson's disease, and homicides (Spellberg et al., 2011). The estimated economic cost of antibiotic resistance ranges from $21 to 34 billion dollars per year (Spellberg et al., 2011). In response, medical practitioners have attempted to reduce the number of inappropriate and unnecessary antibiotic prescriptions. The biomedical research community is also focusing its research efforts to develop new antibiotics as well as alternatives to antibiotic chemotherapy (Kohanski et al., 2010; Jabes, 2011; Edgar et al., 2012). Finally, in Sweden and Switzerland, the use of antibiotics in agriculture for growth promotion and prophylaxis has been banned (Wierup, 2001; Arnold et al., 2004).

Despite these initiatives, a significant body of research suggests that antibiotic resistant bacteria (ARB) are becoming increasingly more prevalent (Palumbi, 2001; Levy and Marshall, 2004; Levy, 2005). An alternative, but complementary, approach to reducing the prevalence of ARB would be to identify pertinent reservoirs of resistance and then to implement appropriate technologies to ameliorate these reservoirs. Consistent with this approach, numerous studies have identified untreated municipal wastewater (raw sewage) as a significant reservoir of ARB and antibiotic resistance genes (ARGs) (Bönemann et al., 2006; da Silva et al., 2006; Auerbach et al., 2007; Schlüter et al., 2007; Szczepanowski et al., 2009; Zhang et al., 2009a; Galvin et al., 2010; Uyaguari et al., 2011; Zhang and Zhang, 2011). Municipal wastewater treatment processes, therefore, should represent an important opportunity to mitigate the quantity of this reservoir of antibiotic resistance.

Although prior research has demonstrated that the treated municipal wastewater also contains substantial concentrations of ARB and ARGs (da Silva et al., 2006; Pruden et al., 2006; Graham et al., 2011; LaPara et al., 2011), a mass balance on wastewater treatment operations suggests that >99% of the ARB and ARGs in untreated municipal wastewater accumulate in the residual wastewater solids. These are subsequently treated by numerous technologies to reduce their nutrient and pathogen content (to varying degrees) prior to their disposal on agricultural land (Tchobanoglous et al., 2003). There have been relatively few investigations on the different technologies used for treating residual wastewater solids and their associated effectiveness at mitigating ARB and ARGs. Diehl and LaPara (2010) observed relatively little removal of ARGs in aerobic digestion processes operated at 22–55°C, but observed increasingly effective removal of ARGs in anaerobic digestion processes at temperature >37°C. In contrast, Ma et al. (2011) observed little benefit of increasing the temperature of anaerobic digestion beyond 37°C.

In the present research, we undertook a detailed investigation of the effectiveness of a bench-scale conventional aerobic digestion process at mitigating the quantity of ARGs in untreated residual wastewater solids. Although our prior research had observed no effect of aerobic digestion on the quantity of ARGs in wastewater solids (Diehl and LaPara, 2010), these previous experiments were performed in relatively small bioreactors with a mean hydraulic residence time of 4 days. Assuming that ARGs decay at a relatively slow rate (i.e., half-lives > 4 days), this experimental design would have been insufficient to observe significant reductions in ARGs. This short time period used in our prior experimental design is also pertinent because the United States Environmental Protection Agency requires that aerobic digestion processes have a mean hydraulic residence time of 40 days (when operated at 20°C) to qualify as a “process to significantly reduce pathogens” (PSRP), which must be achieved before these treated wastewater solids can be applied to agricultural land for their disposal (albeit with some restrictions) (Tchobanoglous et al., 2003). This research is of considerable practical importance because numerous full-scale municipal wastewater treatment facilities currently utilize aerobic digestion processes to treat their wastewater solids, particularly those that treat less than 10 million gallons of wastewater each day (at higher flow rates, other technologies are considered more practical and economical).

## Materials and methods

### Experimental design

A 10-L aerobic digester was operated at room temperature with a mean residence time of 40 days and a minimum dissolved oxygen (DO) concentration of 2 mg/L. The digester was inoculated with 10 L of untreated residual municipal wastewater solids from a full-scale municipal wastewater treatment plant. Mixing and aeration were provided by pumping atmospheric air through a stone diffuser located at the bottom of the reactor vessel at a rate sufficient to prevent settling of solids and to maintain the minimum DO concentration. Typical operating variables, including temperature, DO, pH, total solids, volatile solids, and inert solids, were monitored throughout the entire time period the digester was in operation (Clesceri et al., 1999). The total solids concentration represents the quantity of material remaining after drying at 103°C (i.e., the sum of inorganic and organic material dissolved and suspended in the sample). In contrast, the volatile solids concentration represents the fraction of the total solids concentration that is lost upon ignition at 550°C, whereas the IS concentration represents the fraction of the total solids that is not lost upon ignition at 550°C (i.e., the fraction of the total solids that represents that ash-material). Water loss from the digestor due to evaporation was monitored and replaced by adding appropriate volumes of deionized water to the digester.

The digester was operated for more than 175 days while being fed on a weekly basis untreated residual municipal wastewater solids. The digester was considered to have reached steady-state conditions once the residence time for inert solids (i.e., the average amount of time that an “inert solid” would reside in the aerobic digester) had been maintained at 41.1 ± 0.5 days (mean ± standard deviation) for a time period of 35 days. Once steady-state conditions had been established, the operating mode of the digester was shifted to better reflect continuous-flow operating conditions by feeding untreated residual municipal wastewater solids on a daily basis from Day 180 to Day 191. Following this semi-continuous flow phase, the aerobic digester was operated for an additional 27 days while being fed on a weekly basis untreated residual municipal wastewater solids. On Day 218, half of the digester contents (i.e., 5 L) were replaced with untreated residual municipal wastewater solids to allow the determination of decay coefficients in a batch-like reactor.

### Sample collection and genomic DNA extraction and purification

Triplicate samples (100 μL) were collected from larger aliquots (50–300 mL) of digester contents to ensure accurate sample collection volumes. Samples were then diluted with 500 μL of lysis buffer (120 mM sodium phosphate buffer, 5% dodecyl sulfate, pH 8.0 ± 0.1) and subjected to three consecutive freeze-thaw cycles followed by incubation at 70°C for 90 min. Genomic DNA was then extracted using a FastDNA Spin Kit (MP Biomedicals LLC, Solon, OH) according to the manufacturer's instructions.

### Real-time PCR

Real-time PCR was used to quantify the concentrations of three different genes that encode resistance to tetracycline [*tet*(A), *tet*(W), and *tet*(X)], one gene that encodes resistance to erythromycin [*erm*(B)], one gene that encodes resistance to sulfonamides (*sul1*), and the integrase gene of class 1 integrons (*intI1*). The three tetracycline resistance genes were selected because they represent each of the three known mechanisms of tetracycline resistance (efflux pumps, ribosomal protection proteins, and enzymatic modification) (Levy et al., 1999). The erythromycin gene, *erm*(B), was chosen because it encodes an rRNA methyltransferase that confers resistance to macrolides, lincosamides, and streptogramin B (Roberts et al., 1999). Prior work has demonstrated that all five of these ARGs are present at substantial concentrations in wastewater and/or wastewater solids (Diehl and LaPara, 2010; LaPara et al., 2011; Munir et al., 2011). Class 1 integrons were quantified because of their association with multiple antibiotic resistance. These integrons enable bacteria to collect multiple, exogenous ARGs and modulate their expression (Mazel, 2006). qPCR was also used to determine the concentrations of 16S rRNA genes (a measure of total bacterial biomass), all *Bacteroides* spp. (a measure of total fecal bacteria), and human-specific *Bacteroides* spp. (a measure of human fecal bacteria) (Muyzer et al., 1993; Bernhard and Field, 2000; Layton et al., 2006). Additional information regarding the use of qPCR to quantify these genes can be found in Table 1.

**Table 1:** **Gene targets, resistance mechanisms, primer sequences, amplicon sizes, and annealing temperatures for real-time PCR assays**.

**Gene target**	**Resistance mechanism**	**Primer sequence (5′ → 3′)**	**Size (bp)**	**Annealing temperature (°C)**	**References**
16S rRNA gene	NA	F: CCT ACG GGA GGC AGC AG	202	60	Muyzer et al., 1993
		R: ATT ACC GCG GCT GCT GG			
*Bacteroides* spp.	NA	F: GAG AGG AAG GTC CCC CAC	116	60	Layton et al., 2006
		R: CGC TAC TTG GCT GGT TCA G			
Human-specific *Bacteroides*	NA	F: ATC ATG AGT TCA CAT GTC CG	82	56	Bernhard and Field, 2000; Seurinck et al., 2005
		R: TAC CCC GCC TAC TAT CTA ATG			
*erm*(B)	Ribosomal protection	F: GAT ACC GTT TAC GAA ATT GG	364	58	Chen et al., 2007
		R: GAA TCG AGA CTT GAG TGT GC			
*intI1*	Class 1 integron	F: CCT CCC GCA CGA TGA TC	280	60	Goldstein et al., 2001
		R: TCC ACG CAT CGT CAG GC			
*sul1*	Enzymatic modification	F: CCG TTG GCC TTC CTG TAA AG	67	60	Heuer and Smalla, 2007
		R: TTG CCG ATC GCG TGA AGT			
*tet*(A)	Efflux	F: GCT ACA TCC TGC TTG CCT TC	210	60	Ng et al., 2001
		R: CAT AGA TCG CCG TGA AGA GG			
*tet*(W)	Ribosomal protection	F: GAG AGC CTG CTA TAT GCC AGC	168	60	Aminov et al., 2001
		R: GGG CGT ATC CAC AAT GTT AAC			
*tet*(X)	Enzymatic modification	F: AGC CTT ACC AAT GGG TGT AAA	278	60	Ghosh et al., 2009
		R: TTC TTA CCT TGG ACA TCC CG			

Real-time PCR was carried out on an Eppendorf Mastercycler EP Realplex thermal cycler (Eppendorf, Westbury, NY). PCR assays were optimized to reduce or eliminate the formation of primer-dimers and non-specific products. Typical PCR assays began with a 1 min initial denaturation at 95°C. This step was followed by 40 cycles of denaturation at 95°C for 15 s and combined annealing and extension at the primer-specific annealing temperature for 1 min. Typical reaction volumes were 25 μL and consisted of 12.5 μL of BioRad iTaq SYBR Green Supermix with ROX (Life Science Research, Hercules, CA), 25 μg of bovine serum albumin, optimized quantities of forward and reverse primers, and approximately 1 ng of template genomic DNA. Each analysis consisted of three replicates. Standards were made from PCR products that targeted specific genes from either well-described bacterial isolates or from municipal wastewater solids. PCR products were ligated into a pGEM-T Easy cloning vector, transformed into JM109 competent cells, and extracted from cell cultures using the alkaline lysis procedure (Sambrook et al., 1989). It was confirmed that all plasmids contained the specified gene by nucleotide sequence analysis of the extracted plasmid. The DNA concentration of plasmid extracts was quantified using a TD-700 fluorometer and Hoechst 33258 dye. Each standard curve consisted of a 10-fold dilution series containing at least 5 standards (*r*^2^ ≥ 0.99). Amplification efficiencies were 100 ± 10%.

### Data analysis

All data obtained from groups of triplicate samples were treated as if they had been obtained from a normal distribution (i.e., means and standard deviations were used to describe the data). This assumption of normal distributions was based on results from Shapiro–Wilk normality tests performed in SigmaPlot 12.0 that indicated the complete semi-continuous flow data series for most gene targets could not be distinguished from a normal distribution (*P* > 0.05). Analysis of variance (ANOVA; Microsoft Excel 2010) was used with data from the semi-continuous flow experiment to determine the statistical significance of differences in gene target concentrations between untreated and treated residual solids samples.

Simple linear regression (Arc 1.06) was used with log-transformed data from the batch experiment to determine the goodness of fit of the data to a first-order kinetic model. The first-order kinetic model was chosen based on previous empirical observations that it tends to fit this type of data well. Values of *P* used to compare the relative statistical significance of different kinetic coefficients (Table 4) were determined using Welch's *t*-test for unequal *n* and unequal sample variance. The sample variance for each estimated first-order kinetic coefficient was obtained from the estimated standard error for that coefficient as provided by Arc 1.06.

## Results

### Semi-continuous flow operating mode

With respect to typically monitored operating variables, the lab-scale aerobic digester performed as an appropriate experimental model simulation during the semi-continuous flow experimental period (Table 2). The pH was circumneutral, digester temperature was the same as the ambient air temperature, and DO concentrations were well above the target DO concentration of 2 mg/L. The total and volatile solids concentrations for the untreated and treated solids were well within ranges typically encountered in practice (2–5% total solids, 0.6–3% volatile solids) (Tchobanoglous et al., 2003). The fractions of total and volatile solids destroyed were 26 and 41%, respectively, which is also typical for a full-scale aerobic digestion process (Tchobanoglous et al., 2003).

**Table 2:** **Operating variables during operation in semi-continuous flow mode**.

**Variable**	**Mean ± SD**	***n* values**
pH	7.5 ± 0.2	*n = 12*
Temperature (°C)	17.0 ± 0.3	*n = 12*
Dissolved oxygen (mg/L)	5.8 ± 1.5	*n = 12*
Hydraulic residence time (days)	13.5 ± 0.7	*n = 12*
Untreated total solids	4.6% ± 0.04%	*n = 4*
Treated total solids	3.4% ± 0.3%	*n = 4*
Total solids destruction	26.3% ± 6.0%	*n = 4*
Total solids residence time (days)	33.1 ± 0.4	*n = 12*
Untreated volatile solids	3.2% ± 0.05%	*n = 4*
Treated volatile solids	1.9% ± 0.1%	*n = 4*
Volatile solids destruction	41.1% ± 4.8%	*n = 4*
Volatile solids residence time (days)	27.7 ± 0.6	*n = 12*
Untreated inert solids	1.5% ± 0.02%	*n = 4*
Treated inert solids	1.5% ± 0.1%	*n = 4*
Inert solids destruction	−5.7% ± 8.4%	*n = 4*
Inert solids residence time (days)	41.7 ± 0.1	*n = 12*

**Figure 1:**
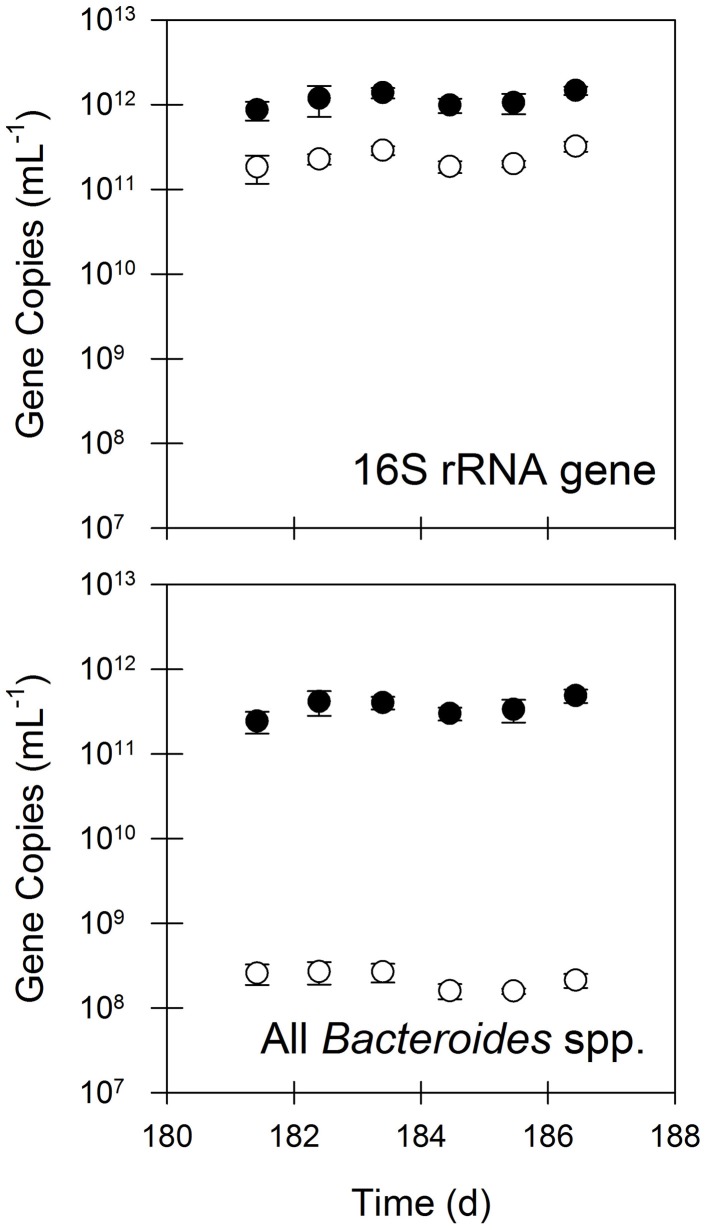
**The quantities of 16S rRNA genes and fecal indicator bacteria as measured by 16S rRNA genes of all *Bacteroides* spp. in untreated (closed circles) and treated (open circles) residual solids.** Values are the arithmetic mean of triplicate samples; error bars represent one standard deviation. The concentrations of human-specific *Bacteroides* spp. were approximately 5 × 10^8^ gene copies mL^−1^ in untreated samples, but were below the detection limit (1×10^8^ gene copies mL^−1^) in treated samples.

The aerobic digester eliminated a substantial fraction of bacterial biomass and fecal indicator bacteria (FIB) as measured by qPCR targeting the 16S rRNA gene and 16S rRNA genes specific for all *Bacteroides* spp. and for human-specific *Bacteroides* spp. (Figure 1). The concentrations of 16S rRNA genes were 77% lower in treated samples compared to the untreated samples. This indicates net destruction of total bacterial biomass in the digester, consistent with the total and volatile solids removal. Significant removal was observed for both types of FIB. The concentrations of all *Bacteroides* spp. were 99.9% lower in treated samples compared to untreated samples. Similarly, the concentrations of human-specific *Bacteroides* spp. were approximately 5×10^8^ gene copies mL^−1^ in untreated samples, but were below the detection limit (1×10^8^ gene copies mL^−1^) in treated samples.

The untreated wastewater solids contained substantial quantities of each of the ARGs investigated in this study. The quantities of *intI1, sul1*, and *tet*(W) were similar, present at a concentration of approximately 10^10^ gene copies mL^−1^. In contrast, the concentration of *erm*(B) was approximately 10^11^ gene copies mL^−1^, and the concentrations of *tet*(A) and *tet*(X) were approximately 10^9^ gene copies mL^−1^. Given that the concentrations of 16S rRNA genes were approximately 10^12^ gene copies mL^−1^ in the untreated solids, the ratio of the various antibiotic resistance determinants examined in this study to bacterial cells ranged from approximately 0.1% for *tet*(A) and *tet*(X) to 1% for *intI1, sul1*, and *tet*(W), and to 10% for *erm*(B).

**Figure 2:**
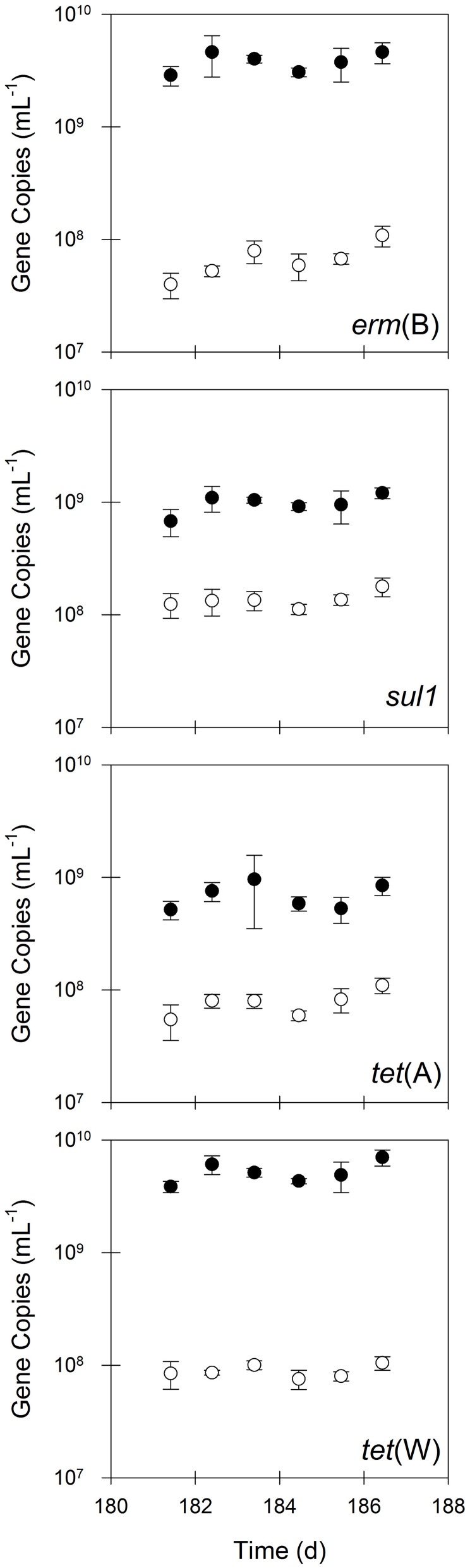
**The quantities of *erm*(B), *sul1, tet*(A), and *tet*(W) in untreated (closed circles) and treated (open circles) residual solids.** Values are the arithmetic mean of triplicate samples; error bars represent one standard deviation.

**Figure 3:**
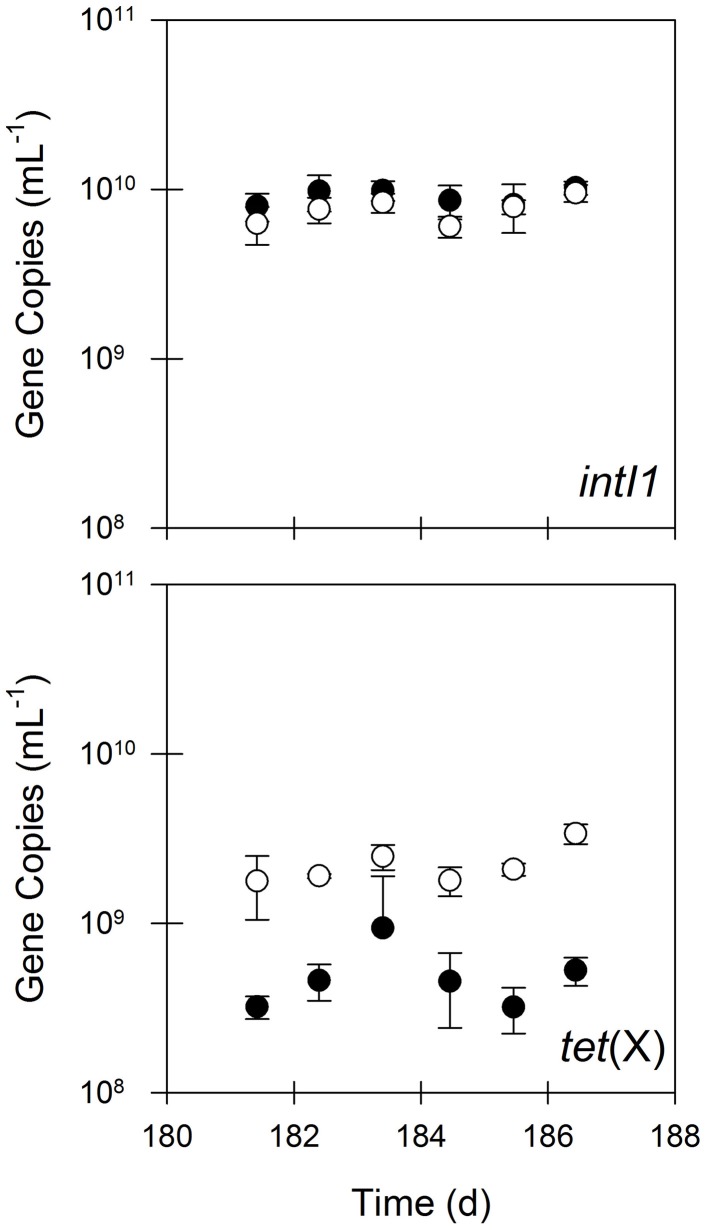
**The quantities of *intI1* and *tet*(X) in untreated (closed circles) and treated (open circles) residual solids.** Values are the arithmetic mean of triplicate samples; error bars represent one standard deviation.

The bench-scale aerobic digester removed between 85 and 98% of *erm*(B), *sul1, tet*(A), and *tet*(W) during the semi-continuous flow experimental period (Figure 2), which was substantially greater than that for bacterial biomass (i.e., 16S rRNA genes). In contrast, the quantity of *intI1* was not statistically different (*P* = 0.17) in the untreated and treated solids, suggesting that aerobic digestion operated in semi-continuous flow mode does not eliminate *intI1* (Figure 3). Furthermore, the ratio of *intI1* to 16S rRNA genes increased in the treatment process from 0.8 to 3%, indicating that aerobic digestion likely selects for bacterial cells possessing a class 1 integron. Interestingly, the aerobic digestion process also appeared to select for bacterial cells containing *tet*(X), as the quantity of this gene was 5-fold greater in the treated solids than in the untreated solids.

### Batch operating mode

Following the semi-continuous flow experimental phase, the aerobic digester was shifted to batch mode to determine decay rates for each target gene. As with the previous experimental phase, typically monitored operating variables indicated that the digester operated as an appropriate simulation of a full-scale aerobic digester. The pH rose from a semi-continuous phase value between 7 and 7.5 to just above 8 following the addition of untreated residual solids, but then gradually decreased to between 7 and 7.5. A substantial decrease in DO concentration to less than 1 mg/L was initially observed, but increased to > 4 mg/L within 24 h and remained so for the duration of the batch experiment.

**Figure 4:**
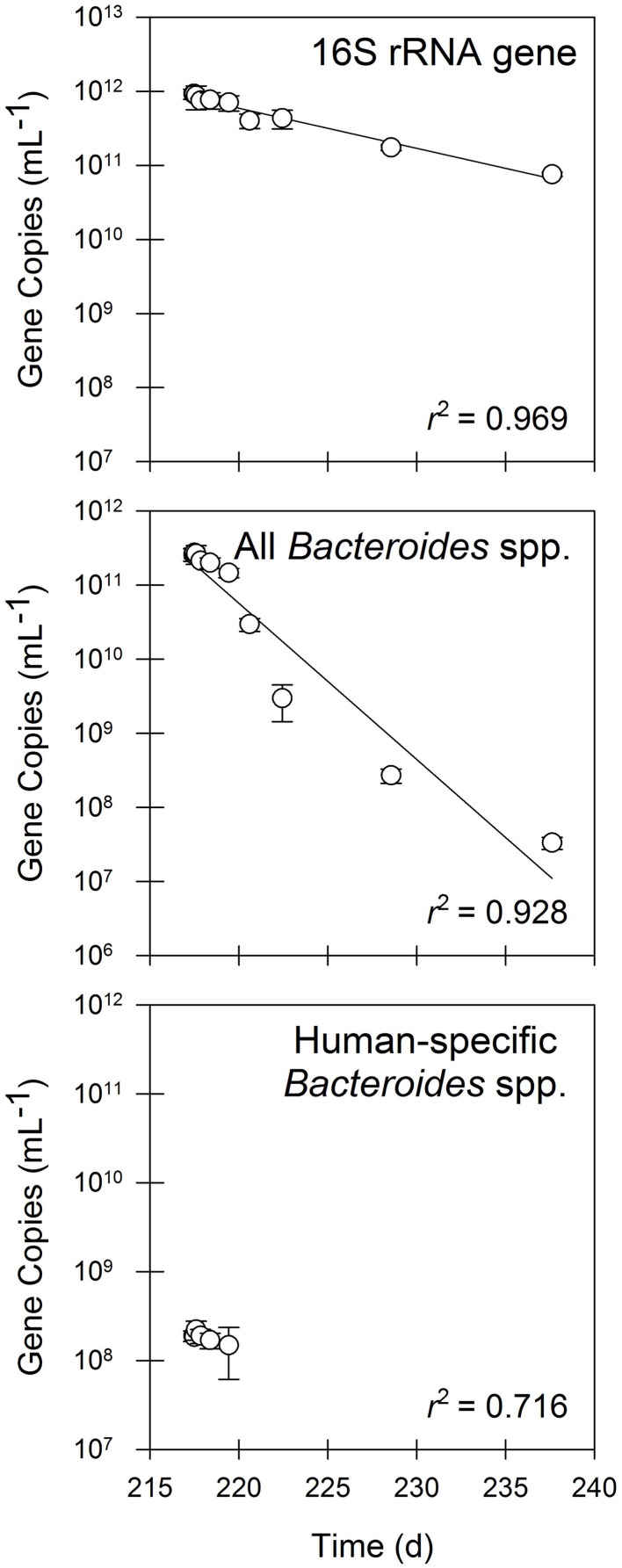
**The quantities of 16S rRNA genes, fecal indicator bacteria as measured by 16S rRNA genes of all *Bacteroides* spp., and fecal indicator bacteria as measured by 16S rRNA genes of human-specific *Bacteroides* spp. in residual solids undergoing batch treatment.** Values are the arithmetic mean of triplicate samples; error bars represent one standard deviation.

A significant decay rate was observed for 16S rRNA genes and FIB during operation in batch mode (Figure 4). The quantities of 16S rRNA genes decayed by 90% during the 20-day batch experiment (*t*_1/2_ = 5.5 d; Table 3). In contrast, all *Bacteroides* spp. decayed by nearly four orders of magnitude over 20 days (*t*_1/2_ = 1.4 d; Table 3), whereas human-specific *Bacteroides* spp. decayed to below the detection limit within one week of beginning the batch experimental phase (*t*_1/2_ = 4.6 d; Table 3).

**Table 3:** **Summary of first-order degradation kinetic model parameter estimates for the 16S rRNA gene, fecal indicator bacteria as measured by 16S rRNA genes of all *Bacteroides* spp., fecal indicator bacteria as measured by 16S rRNA genes of human-specific *Bacteroides* spp., *erm*(B), *intI1, sul1, tet*(A), *tet*(W), and *tet*(X) during batch mode operation**.

**Gene target**	***k* (day^−1^) ± standard error (day^−1^)**	***t***_**1/2**_ **(days)**
16S rRNA gene	0.13 ± 0.008	5.5
*Bacteroides* spp.	0.49 ± 0.048	1.4
Human-specific *Bacteroides* spp.	0.15 ± 0.047	4.6
*erm*(B)	0.19 ± 0.025	3.6
*intI1*	0.11 ± 0.011	6.3
*sul1*	0.15 ± 0.009	4.6
*tet*(A)	0.16 ± 0.011	4.4
*tet*(W)	0.25 ± 0.025	2.8
*tet*(X)	0.12 ± 0.006	5.7

All rates were regressed from 10 data points (except human-specific Bacteroides spp., n = 6) and are statistically significant (P < 0.05).

**Figure 5:**
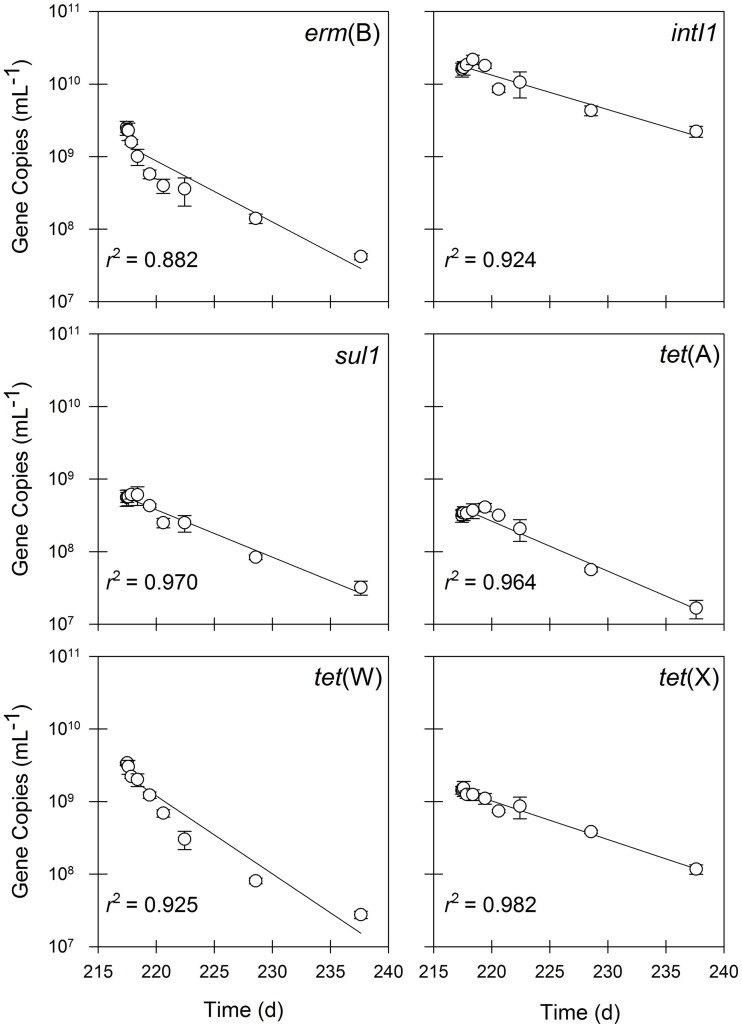
**The quantities of *erm*(B), *intI1, sul1, tet*(A), *tet*(W), and *tet*(X) in residual solids undergoing batch treatment.** Values are the arithmetic mean of triplicate samples; error bars represent one standard deviation.

In contrast to operation in semi-continuous flow mode, the quantities of all of the antibiotic resistance determinants, including *intI1* and *tet*(X), declined in the batch experimental phase (Figure 5). The quantities of *erm*(B) and *tet*(W) declined by approximately two orders of magnitude during the 20-day experiment, whereas the quantities of *intI1, sul1, tet*(A), and *tet*(X) each declined by one order of magnitude during the same time period. Correspondingly, the first-order decay rates varied considerably among individual gene targets. The *intI1* and *tet*(X) genes decayed the most slowly, each with a half-life of approximately 6 days (Table 3). These rates of decay were statistically similar to each other as well as to the rate of decay for the 16S rRNA gene (Table 4). In contrast, the first-order decay rates were significantly (*P* < 0.05) more rapid for the remaining gene targets, with half-lives ranging from 2.8 to 4.6 days (Table 3). These rates of decay were significantly faster than the decay rate for 16S rRNA genes (Table 4).

**Table 4:** **Values of *P* for comparing the relative statistical significance of different kinetic coefficients determined using Welch's *t*-test for unequal *n* and unequal sample variance**.

**Gene**	**16S rRNA gene**	***Bacteroides* spp.**	**Human-specific *Bacteroides* spp.**	***erm*(B)**	***intI1***	***sul1***	***tet*(A)**	***tet*(W)**	***tet*(X)**
16S rRNA gene	1	3 × 10^−5^	0.6	0.02	0.3	0.06	0.03	7 × 10^−4^	0.8
*Bacteroides* spp.		1	2 × 10^−4^	1 × 10^−4^	2 × 10^−5^	5 × 10^−5^	6 × 10^−5^	6 × 10^−4^	3 × 10^−5^
Human-specific *Bacteroides* spp.			1	0.4	0.4	1	0.9	0.1	0.6
*erm*(B)				1	9 × 10^−3^	0.1	0.2	0.1	0.02
*intI1*					1	0.01	5 × 10^−3^	2 × 10^−4^	0.3
*sul1*						1	0.6	4 × 10^−3^	0.02
*tet*(A)							1	6 × 10^−3^	0.01
*tet*(W)								1	6 × 10^−4^
*tet*(X)									1

## Discussion

The long-term goal of our research is to determine how the numerous technologies used to treat municipal wastewater can simultaneously be used to eliminate the substantial quantities of ARB and ARGs that are known to exist in untreated sewage. The present study makes an important advance in our knowledge by elucidating the extent and rate by which aerobic digestion can be used to eliminate ARGs. This research is practically important because the overwhelming majority of ARB and ARGs in raw sewage ultimately end up in the residual wastewater solids, and a recent study suggested that more than 2200 municipal wastewater treatment facilities use this technology to produce more than 85,000 dry tons of treated wastewater solids in the United States each year (Beecher et al., 2007).

In most cases, the rate of disappearance of different ARGs exceeded that of the total number of bacteria (as measured by 16S rRNA gene copies), suggesting that these ARGs were actively eliminated during the aerobic digestion process. Although there is only very limited data presently available in the published literature, these disappearance rates are generally similar to the rates that were previously observed during anaerobic digestion at 37°C (Diehl and LaPara, 2010). In contrast, the quantities of *tet*(X) and *intI1* decayed at a rate similar to that of all bacteria, suggesting that these genes were passively eliminated, paralleling the decline in the total number of bacteria. This observation is substantially different than our previous study, in which both *tet*(X) and *intI1* rapidly declined in bench-scale anaerobic digestion processes operated at temperatures of 37°C or higher (Diehl and LaPara, 2010). Therefore, the rates by which different ARGs decay in a conventional aerobic digestion process are either similar to or slower than the decay rates observed in anaerobic digestion processes. This apparent inferiority of aerobic digestion is pertinent because anaerobic digestion is also a commonly used technology to treat residual wastewater solids (Tchobanoglous et al., 2003).

This research also demonstrated that reactor design has a major effect on the fate of ARGs during the treatment of residual municipal wastewater solids. The most obvious difference occurred with *tet*(X), which declined under batch experimental conditions but increased substantially in semi-continuous flow conditions. Similarly, the quantity of *intI1* declined under batch conditions but remained static during semi-continuous flow operation. In contrast, model results suggest that the quantities of *erm*(B) and *tet*(W) declined more rapidly in semi-continuous flow operation than would be suggested by the first-order decay coefficient elucidated under batch conditions (analyses not shown). Previous researchers have suggested that reactor design affects the removal of ARGs during wastewater solids digestion (Ma et al., 2011); our results support this hypothesis. Additional research is needed to clarify the importance of reactor design and the disappearance of ARGs during the digestion of wastewater solids.

A growing body of evidence suggests that class 1 integrons, which are linked to multiple antibiotic resistance, are particularly prominent in wastewater (Ghosh et al., 2009; Zhang et al., 2009b; Ramsden et al., 2010; Ma et al., 2011; Stalder et al., 2012). Our research has shown significant variation in removal efficiencies of *intI1* depending on the specific technology (i.e., anaerobic digestion achieves better and more efficient removal than aerobic digestion) and the specific operating conditions (temperature, flow regime, etc.). Additional research is needed to better understand the fate and gene cassette content of class 1 integrons in residual solids treatment systems.

Prior research has suggested that *sul1* flanks all class 1 integrons (Mazel, 2006). When the aerobic digester was operated in semi-continuous flow mode, however, the quantities of *sul1* genes declined by almost an order of magnitude, whereas the quantities of *intI1* were similar in the treated and in the untreated residual solids. Similarly, the rate by which *sul1* declined in batch operating mode was significantly faster than the rate by which *intI1* declined (*P* = 0.01; Table 4). This suggests that the coupling of the *sul1* gene to class 1 integrons is not universal; additional research is needed to better understand the relationship between class 1 integrons and *sul1* genes in the unit operations used to treat wastewater solids.

The most significant limitation of our research is the use of real-time quantitative PCR targeting various ARGs as a surrogate for ARB. The genes quantified here could be present in dead but intact bacteria or in bacteria in which the gene is non-functional. Similarly, the identity of the ARB harboring the ARGs detected in this study, and their clinical significance, remain unknown. Finally, only a select group of ARGs were targeted; even though these genes represent several important classes of antibiotics and all three known molecular mechanisms of resistance to tetracyline, they cover a relatively small cross-section of possible resistance gene targets.

In conclusion, aerobic digestion can be used to eliminate ARGs in untreated wastewater solids, but rates can vary substantially depending on the reactor design and the specific ARG examined. This information represents a critical step toward our long-term goal of applying wastewater treatment technologies to mitigate the spread of antibiotic resistance. This knowledge is particularly useful to wastewater treatment engineers as they compare the relative merits of alternative residual solids treatment technologies and for designing specific unit operations to eliminate ARGs. Specifically, aerobic digestion technology, which is used by numerous full-scale municipal wastewater treatment facilities, appears less effective at eliminating ARGs than both conventional and high temperature anaerobic digestion.

### Conflict of interest statement

The authors declare that the research was conducted in the absence of any commercial or financial relationships that could be construed as a potential conflict of interest.
